# Celastrol and Melatonin Modify *SIRT1*, *SIRT6* and *SIRT7* Gene Expression and Improve the Response of Human Granulosa-Lutein Cells to Oxidative Stress

**DOI:** 10.3390/antiox10121871

**Published:** 2021-11-24

**Authors:** Rita Martín-Ramírez, Rebeca González-Fernández, Jairo Hernández, Pablo Martín-Vasallo, Angela Palumbo, Julio Ávila

**Affiliations:** 1 Laboratorio de Biología del Desarrollo, UDI de Bioquímica y Biología Molecular and Centro de Investigaciones Biomédicas de Canarias (CIBICAN), Universidad de La Laguna, 38206 La Laguna, Spain; rmartira@ull.edu.es (R.M.-R.); refernan@ull.edu.es (R.G.-F.); pmartin@ull.edu.es (P.M.-V.); 2 Centro de Asistencia a la Reproducción Humana de Canarias, 38202 La Laguna, Spain; jairoh@fivap.com (J.H.); apalumbo@fivap.com (A.P.)

**Keywords:** melatonin, celastrol, sirtuins, oxidative stress, granulosa-lutein cells

## Abstract

An excess of oxidative stress (OS) may affect several physiological processes fundamental to reproduction. SIRT1, SIRT6 and SIRT7 are involved in protection stress systems caused by OS, and they can be activated by antioxidants such as celastrol or melatonin. In this study, we evaluate *SIRT1*, *SIRT6* and *SIRT7* gene expression in cultured human granulosa-lutein (hGL) cells in response to OS inductors (glucose or peroxynitrite) and/or antioxidants. Our results show that celastrol and melatonin improve cell survival in the presence and absence of OS inductors. In addition, melatonin induced *SIRT1*, *SIRT6* and *SIRT7* gene expression while celastrol only induced *SIRT7* gene expression. This response was not altered by the addition of OS inductors. Our previous data for cultured hGL cells showed a dual role of celastrol as a free radical scavenger and as a protective agent by regulating gene expression. This study shows a direct effect of celastrol on *SIRT7* gene expression. Melatonin may protect from OS in a receptor-mediated manner rather than as a scavenger. In conclusion, our results show increased hGL cells survival with melatonin or celastrol treatment under OS conditions, probably through the regulation of nuclear sirtuins’ gene expression.

## 1. Introduction

Oxidative stress (OS) or the imbalance between reactive oxygen species (ROS) and antioxidants, causes damage to proteins through aggregation and/or denaturation, lipid peroxidation and nucleotide changes in the DNA structure [1]. OS may affect many physiological processes, including those involved in reproduction as folliculogenesis, fertilization or implantation [2,3].

Sirtuins are a family of proteins with NAD+-dependent deacylase and/or ADP ribosyltransferase activity [4]. In mammals, this family includes seven sirtuins (SIRT1-SIRT7) that play an important role in many cellular biological processes such as transcriptional regulation, inflammatory response, oxidative stress, cell survival, DNA repair or energy metabolism [5]. Sirtuins share a conserved core catalytic domain but differ in catalytic activities, subcellular localization, protein targets, and biological functions [6].

Nuclear sirtuins include SIRT1, SIRT6 and SIRT7 [7]. SIRT1 shuttles to the cytoplasm to act on cytoplasmic targets [8]. In mouse knockout models, deficiencies of Sirt1, Sirt6, and Sirt7 are associated with premature aging syndromes [9,10,11]. OS is an important factor in inducing cell senescence because it leads to DNA damage or decreased telomerase activity [12]. The peroxisome proliferator activated receptor g (PPAR-g) is a non-histone protein target of SIRT1 and plays a role in the antioxidant stress system, inducing the expression of antioxidant enzymes [13]. Deacetylation of FOXO3a increases catalase expression, providing protection from damage caused by OS [14].

SIRT6 plays an important role in genome maintenance and DNA repair [15]. Specifically, under OS conditions, Sirt6 is recruited to DNA damage sites and modulates repair of DNA double strand breaks (DSBs) [16]. SIRT6 maintains genomic and telomeric integrity in mammalian cells through a complex that includes MutY homologue (MYH) DNA glycosylase under oxidative DNA damage [17].

SIRT7 regulates transcription of rDNA interacting with RNA Polymerase I and histones [18]. SIRT7 is also a modulator of stress response by adapting cells to environmental challenges [19,20].

Celastrol is a natural triterpenoid isolate from Tripterygium wilfordii [21] with preventive and therapeutic properties for metabolic dysregulations such as obesity, cancer or inflammatory and neurodegenerative diseases [22,23]. Celastrol also has a protective effect against oxidative effects via activation of NRF2 signaling pathway [24] or decreasing lipid synthesis in the liver and improving anti-oxidative status by increasing *SIRT1* expression [25].

Melatonin is a hormone synthesized by a wide variety of animal cells and tissues [26] with antioxidant and free radical scavenger activity [27,28]. There is evidence that melatonin as an anti-oxidative regulator, plays a role in the reproductive system during oocyte maturation [29], steroidogenesis capacity acquisition [30] or embryo implantation [31]. In addition, melatonin has been involved in the clearance of free radicals in oocytes during ovulation [32]. Melatonin effects could be mediated by sirtuins because its action seems to be associated with SIRT1 upregulation [33] and activation of SIRT6 and AMPK-PGC-1α-AKT pathways of signaling after long-term melatonin administration [34].

The aims of this study were to determine the expression of nuclear sirtuins, *SIRT1*, *SIRT6* and *SIRT7*, in response to oxidative stress induced by glucose or peroxynitrite in cultured human granulosa-lutein cells and to evaluate the effect of the antioxidants, celastrol and melatonin as protective agents.

## 2. Materials and Methods

### 2.1. Subjects

Human granulosa-lutein (hGL) cells were obtained from 109 healthy women between 18 and 27 years of age participating in an oocyte donation program (OD). All the procedures and the informed consent from patients were approved by the Ethics Committee of the Universidad de La Laguna (CEIBA2012-0044).

### 2.2. Ovulation Induction Protocol

Ovulation induction was performed using recombinant FSH (Gonal F, Serono, Madrid, Spain), combined with recombinant LH (Luveris, Serono, Madrid, Spain) or human menopausal gonadotropins (Menopur, Ferring, Madrid, Spain) [35]. The hormonal doses administered to each patient were adjusted according to their individual response. Mature oocytes and follicular fluid (FF) were obtained by ultrasound-guided egg retrieval 36 h after the administration of 0.4 mg of leuprolide acetate (Procrin solution, Abbvie, Madrid, Spain).

### 2.3. Isolation of hGL Cells

hGL cells were isolated from FF of each woman by light centrifugation and washed in ‘‘isolation medium’’ (Medium 199, supplemented with sodium bicarbonate [3.7 g/L], penicillin [59 mg/L], streptomycin [100 mg/L], amphotericin B [25 mg/L], L-glutamine [0.29 g/L], and bovine serum albumin [0.1%]). Red blood cells and leukocytes were removed by 50% Percoll gradient and anti-CD45-coated magnetic beads (Dynabeads M-450 CD45; Dynal ASA, Oslo, Norway), respectively. Cellular viability (minimum 95%) was confirmed by trypan blue exclusion test.

### 2.4. Cell Culture and Treatments

Approximately 2.5 × 10^5^ viable cells per well were plated in sterile 6-well dishes (Thermo Fisher Scientific, New York, NY, USA) and cultured for 48 h at 37 °C under 5% CO_2_ in McCoy’s 5A medium supplemented with l-glutamine (0.29 g/L), BSA (0.1%), penicillin (59 mg/L), streptomycin (100 mg/L), and amphotericin (25 mg/L).

#### 2.4.1. Glucose Treatment

In 17 cell cultures, glucose was added alone or in combination with FSH (added after 24 h) according to the following conditions: control, +20 mM glucose, +20 mM glucose + 100 ng/mL FSH, +100 ng/mL FSH.

#### 2.4.2. Peroxynitrite Treatment

Peroxynitrite were added after 19 h to 20 cell cultures according to the following conditions: control, +0.1 mM peroxynitrite, +0.1 mM peroxynitrite + 100 ng/mL FSH, +100 ng/mL FSH. Peroxynitrite was incubated for 10 min and then fresh medium was added. FSH was added after the first 24 h of culture.

#### 2.4.3. Antioxidant Treatment

Glucose and peroxynitrite treatments were replicated with the addition of celastrol 1 μM (glucose + celastrol *n* = 19; peroxynitrite + celastrol *n* = 18) or melatonin 10 μg/mL (glucose + melatonin *n* = 18, peroxynitrite + melatonin *n* = 17). The effect of celastrol or melatonin alone was tested in the same donors.

### 2.5. Gene Expression Analysis by qRT-PCR

Forty-eight hours after seeding, total RNA from each cell culture was isolated using Aurum total RNA mini kit (Bio-Rad Laboratories, Hercules, CA, USA) and reverse transcribed using iScript cDNA Synthesis kit (Bio-Rad Laboratories, Hercules, CA, USA), adding 1 µg RNA per reaction, following the manufacturer’s instructions. Relative gene expression was performed using the following primers: *SIRT1* (CTATACCCAGAACATAGACACG, ACAAATCAGGCAAGATGC), *SIRT6* (AGGGACAAACTGGCAGAGC, TTAGCCACGGTGCAGAGC) and *SIRT7* (GCAGAGCAGACACCATCC, GTTCACGATGTAAAGCTTCG) and β-actin (CTTCCTTCCTGGGCATGG, GCCGCCAGACAGCACTGT) as a housekeeping gene. All amplification reactions were carried out in a BioRad CFX96 real-time PCR system (Bio-Rad Laboratories, Hercules, CA, USA) and performed with 2× Sso Fast Eva Green Supermix (Bio-Rad Laboratories, Hercules, CA, USA) and 0.4 µmol/L of each primer in a final volume of 10 µL.

All samples were analyzed in triplicate using the following thermal profile: 30 s at 95 °C, 45 cycles at 95 °C for 5 s plus 59 °C for 5 s. The melting curve program was performed at 65 °C to 95 °C with a heating rate of 0.1 °C/s and read every 0.5 °C. Gene expression levels are presented as individual data points using the mean of triplicates to calculate 2^ΔCT^ [36].

### 2.6. DNA Damage Assay

Isolated hGL cells were seeded on poly l-lysine (Sigma, St. Louis, MO, USA) pre-coated 15 mm coverslips and fixed in methanol 100% for 6 min at −20 °C. The blocking step was performed using universal blocking buffer (PBS buffer supplemented with 1% BSA, 0.1% gelatin, 0.5% Triton X-100, 0.05% sodium azide) after washing three times with PBS buffer (Na_2_HPO_4_ 1.09 g, NaH_2_PO_4_ 0.32 g, NaCl 9 g, H_2_O 1000 mL, pH 7.4). Anti-8-OHdG (15A3) antibody (dilution, 1:50; Santa Cruz Biotechnology Inc., Dallas, TX, USA) diluted in blocking solution was incubated for 1 h at room temperature and then washed three times with PBS buffer. Secondary anti-mouse IgG FITC conjugate antibody (dilution, 1:125; Boehringer Mannheim, Baden-Wurttemberg, Germany) was incubated for 1 h. Finally, the coverslips were mounted using ProLong Diamond Antifade Mountant with DAPI (Thermo Fisher Scientific Inc., Waltham, MA, USA) and analyzed using Leica SP8 confocal microscope (Leica Microsystems CMS, Mannheim, Germany). DNA damage was quantified by levels of 8-hydroxy-2′-deoxyguanosine (8-OHdG) staining and cell survival was estimated by DAPI staining in all cell culture conditions as described above. Images were analyzed using Image J 1.53 software (https://imagej.nih.gov/ij/index.html). Cells were counted in 20 different fields and classified in three groups with respect to their fluorescence level: high (cells fluorescent at 100% maximum brightness intensity), medium (cells fluorescent at 60% brightness intensity) and low (cells fluorescent at 20% brightness intensity). DNA damage was calculated as the percentage of cells with different 8-OHdG staining levels in each condition tested. Cell survival was estimated based on the total number of cells (DAPI staining) relative to number of cells in each control.

### 2.7. Oxidative Stress Assay

2′,7′-dichlorofluorescin diacetate (DCFDA), also known as the H2DCFDA-Cellular Reactive Oxygen Species Detection Assay Kit (Abcam, Cambridge, England) was used for measure the cellular OS level. This kit evaluates the oxidation of DCFDA by ROS or reactive nitrogen species (RNS) [37]. OS level was measured in cell cultures with a 25 × 10^3^ cells per well density treated with 10 μg/mL melatonin using 45 μM DCFDA at 24 and 48 h, following the manufacturer’s instructions.

### 2.8. Statistical Analysis

SPSS 23 software (IBM Corporation, Somers, NY, USA) was used to perform the statistical analysis using the Student’s *t*-test to carry out comparisons between each cell culture conditions considering a *p* value of <0.05 as statistically significant. Mean and standard error (SE) are reported.

## 3. Results

### 3.1. DNA Damage in Control and Treated Cells

**Figure 1:**
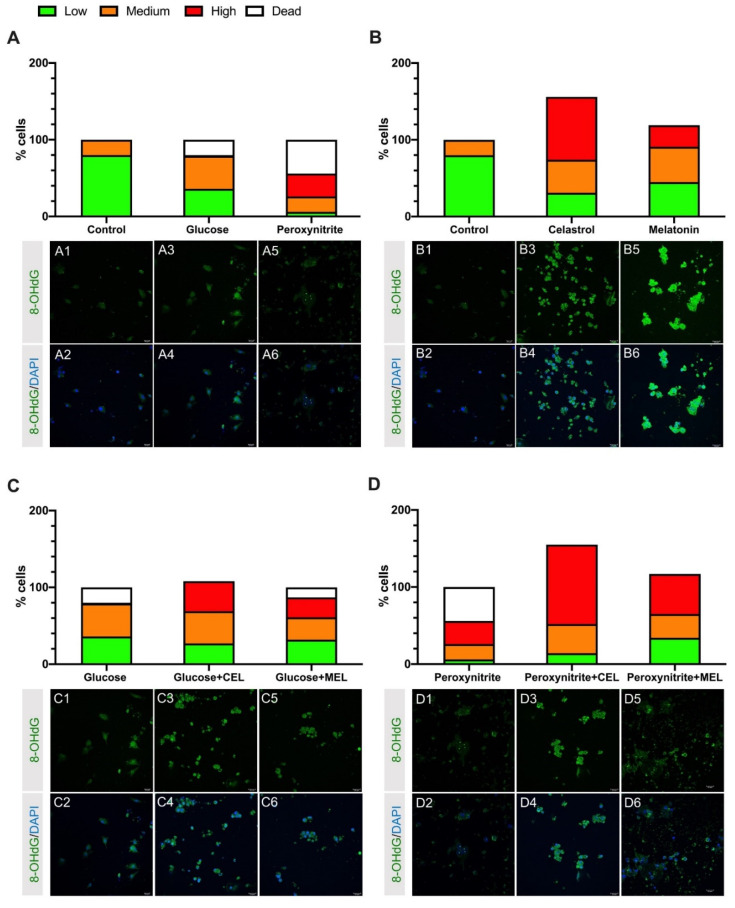
Effect of glucose, peroxynitrite, celastrol (CEL), melatonin (MEL) and their combinations on DNA damage and cell survival in hGL cells. Histograms represent percentage of cells with different 8-OHdG staining intensity defined as low (green), medium (orange) and high (red). Percentage of dead cells is included (white). Representative images of each culture condition are showed below corresponding bar. (**A**) Glucose and peroxynitrite treatments compared to control. (**B**) Celastrol and melatonin compared to control. (**C**) Glucose + CEL and glucose + MEL treatments compared to glucose treatment. (**D**) Peroxynitrite + CEL and peroxynitrite + MEL treatments compared to peroxynitrite treatment. *n* = 3.

Treatment with glucose, and especially, peroxynitrite increased the percentage of the high and medium intensity levels of 8-OHdG staining. Both the number of damaged cells and dead cells increased compared to control cells (Figure 1A).

Human GL cells treated with celastrol or melatonin showed an increase in the high and medium 8-OHdG staining levels compared to control. This increase was higher in celastrol-treated cells. Celastrol and melatonin treatment increased survival of hGL cells by 56% and 20%, respectively (Figure 1B).

Addition of celastrol or melatonin to hGL cells treated with glucose increased the intensity of staining from high and medium 8-OHdG staining levels. Complementarily, the addition of celastrol or melatonin to cell cultures increased survival by 7% or lowered mortality, respectively (Figure 1C). Peroxynitrite-treated cells showed a lower percentage of highly damaged cells, compared to cells treated with a combination of peroxynitrite and celastrol or peroxynitrite and melatonin. Moreover, we found a survival increase of 55% in peroxynitrite + celastrol and 17% in peroxynitrite + melatonin (Figure 1D).

### 3.2. Effect of Melatonin on hGL Cultured Cells

**Figure 2:**
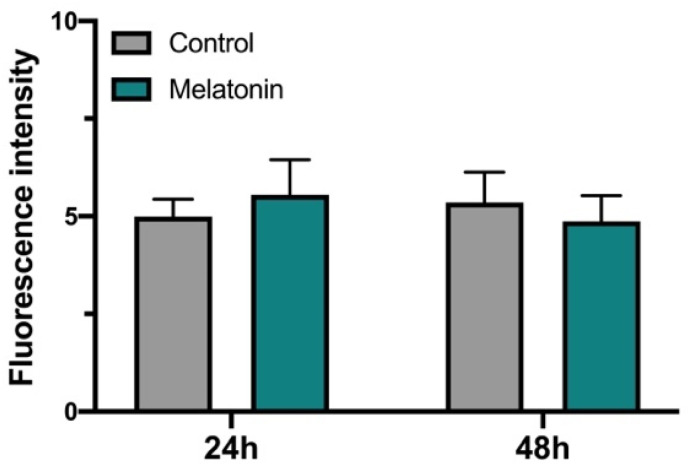
ROS/RNS levels in hGL cells treated with melatonin. Histogram representing total OS measures (*n* = 3) in hGL cells after 24 and 48 h of melatonin exposure compared to control.

ROS/RNS levels in hGL cells treated with melatonin were measured by DCFDA at 24 and 48 h (Figure 2). Statistically significant differences were not found.

### 3.3. SIRT1 Expression in Control and Treated Cells

**Figure 3:**
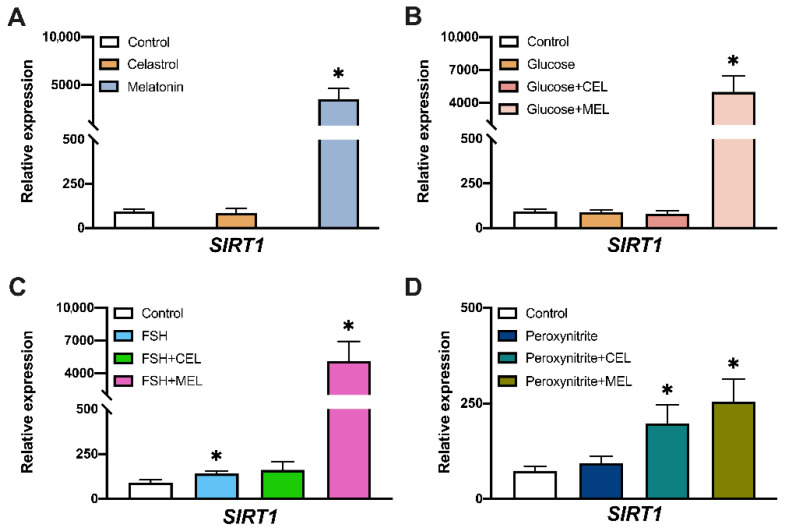
Effect of glucose, peroxynitrite, FSH, celastrol (CEL), melatonin (MEL) and their combinations on *SIRT1* gene expression. *SIRT1* relative expression levels in hGL cells treated with: (**A**) celastrol (*n* = 19) or melatonin (*n* = 18), (**B**) glucose (*n* = 17) and in combination with celastrol (*n* = 19) or melatonin (*n* = 18), (**C**) FSH (*n* = 17) and in combination with celastrol (*n* = 19) or melatonin (*n* = 18), (**D**) peroxynitrite (*n* = 20) and in combination with celastrol (*n* = 18) or melatonin (*n* = 17). Asterisks (*) indicate statistically significant differences compared to control.

Melatonin-treated cells showed an increased gene expression of *SIRT1* compared to control (Figure 3A). The addition of glucose or glucose in combination with celastrol did not modify *SIRT1* mRNA level but the combination of glucose and melatonin increased *SIRT1* expression (Figure 3B). FSH treatment increased *SIRT1* expression compared to control (Figure 3C) but FSH + celastrol addition did not modify gene expression. However, we observed an increased expression of *SIRT1* in cells treated with FSH + melatonin compared to control. Peroxynitrite treatment did not affect *SIRT1* expression but the combination of peroxynitrite with celastrol or melatonin increased *SIRT1* expression (Figure 3D).

### 3.4. SIRT6 Expression in Control and Treated Cells

**Figure 4:**
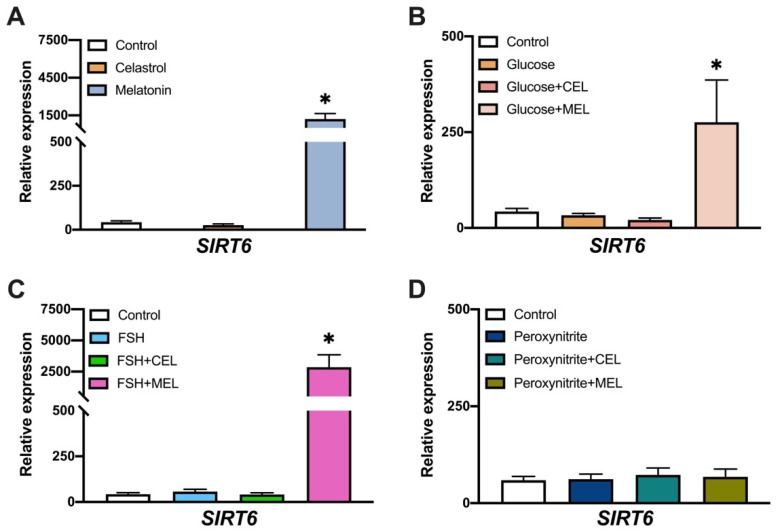
Effect of glucose, peroxynitrite, FSH, celastrol (CEL), melatonin (MEL) and their combinations on *SIRT6* gene expression. *SIRT6* relative expression levels in hGL cells treated with: (**A**) celastrol (*n* = 19) or melatonin (*n* = 18), (**B**) glucose (*n* = 17) and in combination with celastrol (*n* = 19) or melatonin (*n* = 18), (**C**) FSH (*n* = 17) and in combination with celastrol (*n* = 19) or melatonin (*n* = 18), (**D**) peroxynitrite (*n* = 20) and in combination with celastrol (*n* = 18) or melatonin (*n* = 17). Asterisks (*) indicate statistically significant differences compared to control.

Gene expression analysis showed higher *SIRT6* expression in melatonin-treated cells compared to control (Figure 4A). The addition of glucose or FSH, alone or in combination with celastrol, showed no differences in *SIRT6* expression compared to control (Figure 4B,C). However, addition of glucose or FSH combined with melatonin increased *SIRT6* mRNA levels (Figure 4B,C), probably because of the effect of melatonin alone observed before (Figure 4A). Peroxynitrite treatment and combination with celastrol or melatonin did not modify *SIRT6* gene expression compared to control (Figure 4D).

### 3.5. SIRT7 Expression in Control and Treated Cells

**Figure 5:**
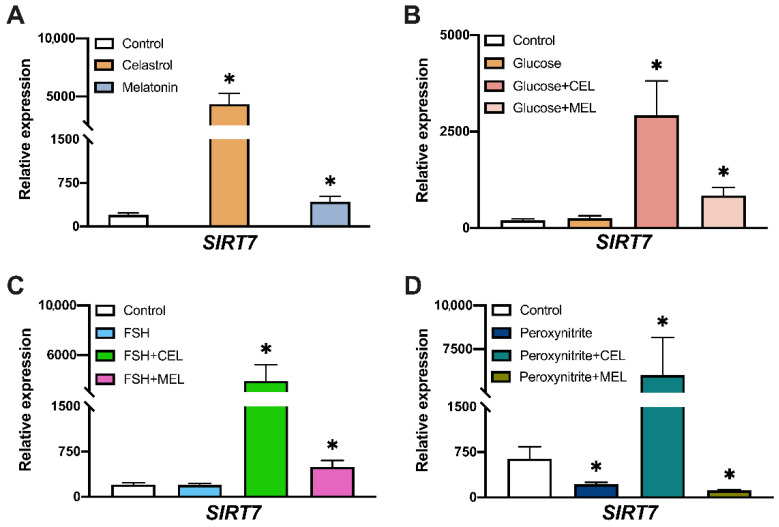
Effect of glucose, peroxynitrite, FSH, celastrol (CEL), melatonin (MEL) and their combinations on *SIRT7* gene expression. *SIRT7* relative expression levels in hGL cells treated with: (**A**) celastrol (*n* = 19) or melatonin (*n* = 18), (**B**) glucose (*n* = 17) and in combination with celastrol (*n* = 19) or melatonin (*n* = 18), (**C**) FSH (*n* = 17) and in combination with celastrol (*n* = 19) or melatonin (*n* = 18), (**D**) peroxynitrite (*n* = 20) and in combination with celastrol (*n* = 18) or melatonin (*n* = 17). Asterisks (*) indicate statistically significant differences compared to control.

The analysis of gene expression showed that the addition of both celastrol and melatonin increased *SIRT7* mRNA levels compared to control (Figure 5A). Glucose or FSH treatment did not modify *SIRT7* expression. However, the combined addition of both celastrol or melatonin increased *SIRT7* mRNA levels (Figure 5B,C), mimicking the effect observed in cells treated with celastrol or melatonin alone (Figure 5A). This effect of celastrol was also observable in peroxynitrite + celastrol (Figure 5D) whereas in peroxynitrite treatment, lower amounts of *SIRT7* mRNA were observed (Figure 5D). *SIRT7* expression also decreased in peroxynitrite + melatonin-treated cells (Figure 5D).

## 4. Discussion

Oxidative stress may be harmful in human reproduction processes and investigation of antioxidant agents that may prevent OS damage in reproductive tissues and processes is of paramount importance to improve both natural fertility and the results of infertility treatments. This research was performed to investigate the effects of celastrol and melatonin in preventing the impact of OS generated by glucose or peroxynitrite on cultured hGL cells.

The addition of OS inductors (glucose or peroxynitrite) to cultured hGL cells increased DNA damage and the percentage of dead cells compared to control, most prominently under peroxynitrite treatment (Figure 1A). Previous studies from our laboratory showed that higher ROS/RNS levels in cells treated with glucose or peroxynitrite [38] increased OS levels, leading to DNA damage and cell death. In this article, we report that the addition of antioxidants (celastrol or melatonin) to culture medium elicits an increase in the amount of high/medium level of 8-OHdG staining (a measure of DNA damage) compared to the amount found in control cells, but in contrast, we found an increase in the total number of surviving cells (Figure 1B). In the case of celastrol, this pro-survival effect could be related to the decrease in ROS/RNS level as upregulation of *SIRT7* gene expression was observed in cultured cells treated with antioxidant [38]. The pro-survival response of melatonin is lower and consistent with the fact that the addition of melatonin to cultured medium did not substantially modify ROS/RNS levels (Figure 2). Interestingly, these results support the role of melatonin as an OS protector in a receptor-dependent manner, instead of a scavenger molecule under the conditions tested here. The effects observed after the addition of celastrol and melatonin were maintained in cells treated with the combination of OS inductor + antioxidant (Figure 1C,D).

Analysis of gene expression in hGL cells treated with glucose showed no variation in sirtuins’ gene expression (Figure 3B, Figure 4B and Figure 5B), even though previous studies demonstrated an increased ROS/RNS by the addition of glucose to culture medium [38]. An increase in DNA damage and in the number of dead cells was observed (Figure 2). Taken together, these data indicate that the addition of glucose increased OS and DNA damage with no variation in sirtuins’ gene expression.

To analyze the effect of antioxidants on hormones that play an important role in oocyte maturation, FSH was added to cultured hGL cells. We observed an increase in *SIRT1* expression in cells treated with FSH, suggesting that SIRT1 could be a mediator of FSH action. To our knowledge, this is the first time that a relationship between FSH and *SIRT1* expression has been described in hGL cells, suggesting a proliferative FSH-effect mediated by SIRT1. This relationship between hFSH and *SIRT1* expression was previously described in porcine ovarian cells [39].

The addition of peroxynitrite decreased *SIRT7* expression levels but did not modify *SIRT1* or *SIRT6* expression (Figure 3D, Figure 4D and Figure 5D). Peroxynitrite is a potent oxidant that produces a high percentage of DNA damage and cell death. *SIRT7* expression is related to cell proliferation and impaired G1/S progression [40]. It is possible that decreased mRNA levels of *SIRT7* in the presence of peroxynitrite induced cell cycle arrest in order to activate DNA repair systems. A post-translational modification of SIRT6 by peroxynitrite-induced OS controlling Sirt6 enzymatic activity has been reported [41], with no alteration in gene expression. This agrees with our data showing the non-variation in SIRT6 expression with peroxynitrite treatment.

Interestingly, we observed changes in gene expression elicited by OS in cultured hGL cells independently of the OS inductor. The addition of melatonin alone or in combination with any of the other substances, induced the expression of the three nuclear sirtuins studied here. The activation of *SIRT1* by melatonin has been widely described in many conditions [42,43,44]. In the mouse ovary, melatonin reduces OS and apoptotic damage by activating SIRT1 signaling in a melatonin receptor-dependent manner [45]. Since our results support a protective effect of melatonin without a reduction in ROS/RNS levels, it is possible that the increased survival observed in hGL cells treated with melatonin could be related to SIRT1 inhibition of apoptosis through Foco1 deacetylation [46]. Previous data also described melatonin-dependent regulation of SIRT6 expression in endothelial cells [47] proposing SIRT6-AMPK-PGC-1α-AKT signaling as a novel downstream target of melatonin membrane receptors [34]. To our knowledge, this is the first report of an effect of melatonin on *SIRT7* gene expression. Since Akt pathway promotes cell survival in response to extracellular signals and a direct role of SIRT7 in Akt regulation has been described [48], it is possible that melatonin could regulate SIRT7 expression through the PI3K/AKT/mTOR pathway similarly to *SIRT6* regulation. This hypothesis is supported by the results of our DNA damage experiments; we found that melatonin treatment increased cell survival (Figure 1B1–B6). The role of SIRT7 in reproduction is not clear, although in female mice, *SIRT7* expression is related to ovarian reserve by playing a role in repairing double-strand DNA breaks [49].

The addition of celastrol, alone or with the addition of OS inductors increased *SIRT7* gene expression under the treatment conditions tested in these experiments. Several studies point to celastrol as a substance with multiple activities such as anti-tumor [50,51], anti-inflammatory [52,53] and antioxidant [54,55], and it participates in cell processes as a molecule affecting signaling of several pathways such as the ERK pathway [56,57]. In this sense, celastrol has been reported to bind Shoc2, a scaffold protein involved in processes such as cell motility, invasion, and proliferation through the ERK pathway [58]. SIRT7 activates ERK1/2 signaling [59]. Taken together, our results may show a compensatory mechanism where *SIRT7* is overexpressed to revert the ERK pathway inhibition by celastrol, leading to maintenance of the cell proliferation status.

In conclusion, our results show that melatonin and celastrol treatments increase hGL cells survival under oxidative stress conditions, probably by regulating the expression levels of nuclear sirtuins. Since sirtuins’ expression is related to the control of several female reproductive processes, modulation of sirtuins’ gene expression may contribute to improved oxidative stress status in normal ovaries and pathologic states.

## 5. Conclusions

Female reproductive functions may be altered by oxidative stress with resulting infertility or pregnancy complications. Research on the ovarian environment under oxidative stress conditions could yield information to prevent female reproduction failure. The aim of this paper was to study the response of nuclear sirtuins to oxidative stress induction and to evaluate the effect of antioxidants. Our results show that treatment with celastrol or melatonin improves oxidative stress effects and both compounds are differently involved in nuclear sirtuins’ expression.

## Data Availability

All data presented in this article can be obtained from Authors, upon request. The data are not publicly available due to privacy and/or ethical restrictions.
